# Supplementary material for: Variation in Bird Eggs—Does Female Factor, Season, and Laying Order Impact the Egg Size, Pigmentation, and Eggshell Thickness of the Eggs of Capercaillie?
Source: Animals (Basel). 2021 Dec 4;11(12):3454. doi: 10.3390/ani11123454 (PMC8697993; doi:10.3390/ani11123454)

Supplementary material

Table S1. Variation of the Capercaillie eggs characteristics (eggshell thickness at the various parts of the egg, mean eggshell thickness, egg size and shape, eggshell durability) in relation to laying order. Correlation were calculated with Pearson coefficient correlation or Spearman correlation coefficient - marked with (S).

Female ID	Thickness at the Blunt end	Thickness at the Equator	Thickness at the Sharp End	Mean Thickness	Eggshell Lightness	Length	Width	Egg Index (Shape)	Durability
				**P-value/Correlation Coefficient Pearson or Spearman (S)**					
1 Blue (n=3)	0.307/0.886	0.124/0.981	0.087/-0.991	**0.004/-1.000**	0.626 0.554	0.716/-0.431	0.476/0.733	0.669/-0.497	*
1/Green (n=4)	0.941/0.093	0.074/0.993	0.207/-0.948	0.864/0.212	**0.042/0.958**	0.231/0.769	0.372/-0.628	0.113/0.887	0.651/0.349
2/Green (n=3)	0.964/-0.056	0.835/-0.256	0.521/-0.683	0.818/-0.282	**0.031/0.999**	0.516/-0.689	0.875/0.195	0.305/-0.888	0.513/-0.692
5/Green (n=3)	0.333/0.866/(S)	0.437/-0.774	0.924/0.119	0.979/0.033	0.228/0.936	0.360/0.845	0.819/0.280	0.157/0.970	0.957/-0.068
6/Red (n=3)	**0.011/1.000**	0.114/0.984	**0.035/0.998**	**0.041/0.998**	0.783/0.335	0.279/-0.905	0.401/0.808	0.108/-0.986	0.333/0.866
6/Green (n=4)	0.606//0.581	0.091/0.990	0.922/-0.122	0.244/0.927	0.455/-0.545	0.939/0.061	0.272/-0.728	0.812/0.188	0.293/-0.896
7/Blue (n=5)	0.242/-0.642	0.458/0.440	0.382/-0.509	0.350/-0.600(S)	0.098/0.808	0.148/0.746	0.157/-0.735(S)	0.142/0.753	0.538/-0.371
8/Blue (n=7)	0.384/-0.392	0.410/-0.373	0.202/-0.549	0.309/-0.452	0.350/-0.419	0.147/-0.609	0.491/-0.315	0.168/-0.585	0.876/-0.073
Józefów (n=3)	0.951/0.077	0.867/-0.208	0.903/0.152	0.951/0.077	0.640/-0.536	0.245/0.927	0.240/0.930	0.259/0.919	0.413/-0.797
Blue (n=7)	0.876/-0.083	0.262/-0.546	0.898/0.068	0.735/-0.178	0.897/0.060	**0.012/-0.867**	0.892/0.064	**0.041/-0.774**	0.147/-0.609
7/Green (n=3)	0.367/-0.839	0.725/-0.418	0.969/-0.049	0.816/-0.285	0.801/0.308	0.705/0.447	0.465/0.745	0.774/0.347	0.212/-0.945
1/Red (n=3)	0.257/0.920	1.000/-0.500(S)	0.818/0.281	0.756/0.373	0.614/0.234	0.925/-0.044	0.599/-0.243	0.920/0.047	0.578/-0.338
Gn/11 (n=5)	0.940/-0.060	0.346/-0.654	0.952/0.048	0.554/-0.446	0.086/-0.824	0.083/-0.900(S)	0.953/-0.037	0.196/-0.692	0.061/-0.995
Cz03 (n=10)	**0.031/-0.678**	**0.035/-0.668**	0.441/-0.275	**0.033/-0.673**	0.701/0.139	**0.025/-0.698(S)**	0.971/0.013	**0.010/-0.794(S)**	*
N01 (n=5)	0.528/-0.380	**0.006/0.972**	*	*	*	*	*	*	*
63 (n=4)	0.933/-0.067	0.822/0.178	*	*	0.863/0.137	0.636/-0.364	0.265/-0.735	0.830/-0.170	0.510/-0.490
56 (n=5)	0.073/-0.842	0.397/-0.494	*	*	1.000/0.000	0.479/-0.422	0.178/-0.712	0.798/-0.159	0.325/-0.561
Gn5 (n=4)	0.446/0.554	0.565/-0.435	*	*	0.653/-0.347	0.856/-0.144	0.389/0.611	0.750/-0.400(S)	0.450/0.550
5/Blue (n=4)	*	*	*	*	*	0.462/0.538	0.056/0.944	*	*
Red (n=3)	*	*	*	*	*	0.618/-0.564	0.098/-0.988	*	0.601/-0.587

Table S2. Variation of eggshells thickness from post-hatched eggshells over the following seasons for the same females. T means performed test was t-test, W means performed test was Mann–Whitney U test, A means performed test was ANOVA

**Female**	**Thickness at the Equator**				**Thickness at the Sharp End**				**Mean Thickness**			
	**Season/Year**			***p*-value**	**Season/Year**			***p*-value**				***p*-value**
	**2018**	**2019**	**2020**		**2018**	**2019**	**2020**		**2018**	**2019**	**2020**	
6 red (n=9)	0.279	0.278	*	0.930 (T)	0.274	0.258	*	0.123 (T)	0.279	0.28	*	0.556 (W)
	0.01	0.023	*		0.006	0.017	*					
9 red (n=10)	0.271	0.259	0.261	0.500 (A)	0.298	0.281	0.265	**0.041 (A)**	0.287	0.27	0.263	0.066 (A)
	0.006	0.015	0.01		0.002	0.003	0.021		0.007	0.008	0.015	
10 red (n=8)	*	0.274	0.279	0.627 (T)	*	0.264	0.266	0.891 (T)	*	0.269	0.269	0.973 (T)
	*	0.006	0.016		*	0.006	0.016		*	0	0.013	
2 green (n=7)	*	0.27	0.262	0.478 (T)	*	0.255	0.253	0.899 (T)	*	0.262	0.259	0.806 (T)
	*	0	0		*	0.012	0.001		*	0	0.016	
5 blue (n=9)	*	0.263	0.268	0.658 (T)	*	0.253	0.268	0.122 (T)	*	0.258	0.267	0.235 (T)
	*	0.017	0.018		*	0.012	0.015		*	0.013	0.014	
8 blue (n=7)	*	0.27	0.267	0.754 (T)	*	0.269	0.268	0.944 (T)	*	0.27	0.267	0.708 (T)
	*	0.012	0.011		*	0.014	0.009		*	0.012	0	
73 (n=8)	*	0.264	0.264	0.997 (T)	*	0.266	0.274	0.359 (T)	*	0.265	0.266	0.897 (T)
	*	0.011	0.007		*	0.018	0.007		*	0.012	0.006	
60 (n=8)	*	0.284	0.288	0.815 (T)	*	0.254	0.287	0.07 (T)	*	0.268	0.287	0.243 (T)
	*	0.024	0.018		*	0.031	0.012		*	0.03	0.013	

Table S3. Capercaillie eggshell characteristics from different breeding centers. T means performed test was t-test, W means performed test was Mann–Whitney U test, A means performed test was ANOVA, K-W means performed test was Kruskall-Wallis test.

	**Unfertile Eggs**				**Post Hatched Eggshells**				
	**Breeding Centre**		**SEM**	***p*-value**	**Breeding Centre**			**SEM**	***p*-value**
	**LF (n=61)**	**WF (n=59)**			**LF (n=74)**	**WF (n=87)**	**GB (n=10)**		
Thickness at the sharp end	0.279	0.284	*	0.873 (W)	0.254	0.262	0.25	*	0.160 (K-W)
Thickness at the equator	0.282	0.280	*	0.441 (W)	0.264	0.272	0.273	*	0.100 (K-W)
Eggshell lightness	68.784<br>3.507	72.310<br>5.345	0.838	**0.037 (T)**	69.438<br>3.908	74.236<br>2.579	67.113<br>1.744	0.766	**0.008 (A)**
Thickness at the blunt end	0.262	0.262	*	0.849 (W)	*	*	*	*	*
Egg shape index	1.367<br>0.070	1.338<br>0.038	0 .009	0.125 (T)	*<br>*	*<br>*	*<br>*	*<br>*	*<br>*
Durability	4.029<br>0.548	3.962<br>0.560	0.093	0.731 (T)	*<br>*	*<br>*	*<br>*	*<br>*	*<br>*
Width	41.037<br>0.583	41.019<br>0.731	0.113	0.937 (T)	*<br>*	*<br>*	*<br>*	*<br>*	*<br>*
Length	56.084<br>2.900	54.882<br>1.939	0.407	0.150 (T)	*<br>*	*<br>*	*<br>*	*<br>*	*<br>*

Figure S1. Coefficient of variation for egg length, width, egg shape index and mean eggshell thickness for particular females in year 2019. The compared unfertilized eggs are marked in blue, the posthatched eggshells are marked in green

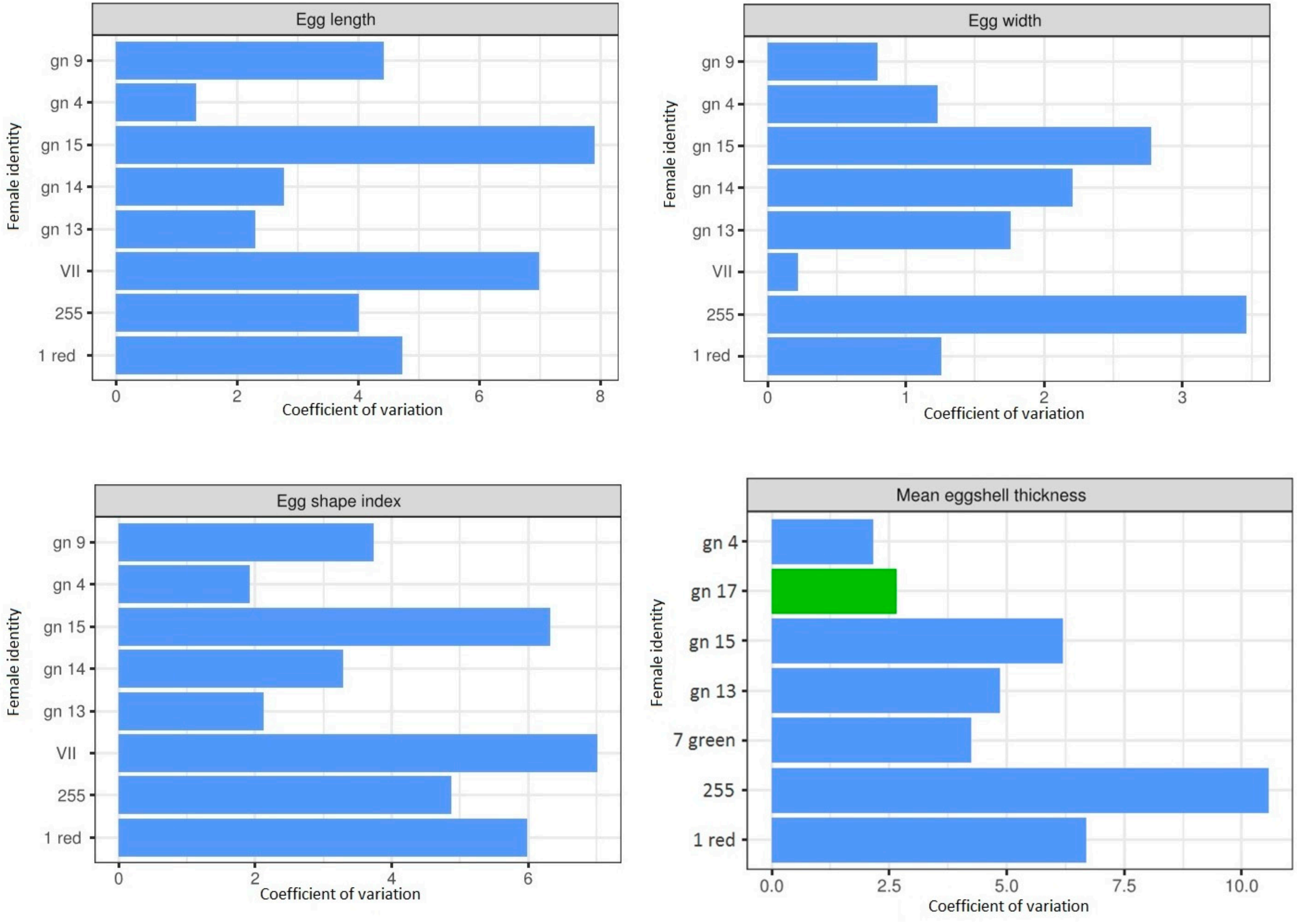

Figure S2. Coefficient of variation for egg length, width, egg shape index and mean eggshell thickness for particular females in year 2020. The compared unfertilized eggs are marked in blue, the posthatched eggshells are marked in green

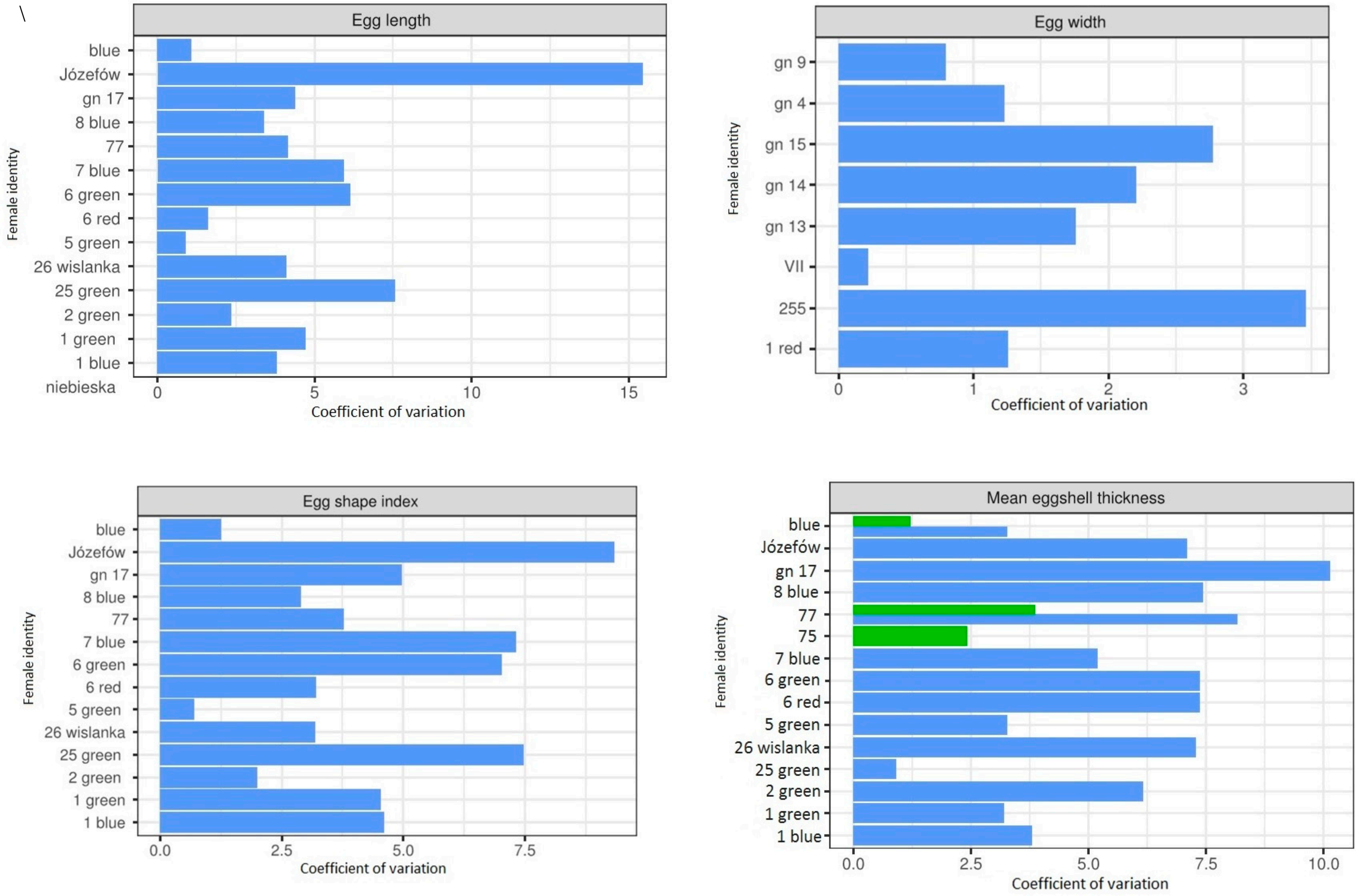

Supplement: Supplementary file 1 [file animals-11-03454-s001.zip › animals-1466467-supplementary.pdf]
